# Complete genome sequence of *Haliscomenobacter hydrossis* type strain (O^T^)

**DOI:** 10.4056/sigs.1964579

**Published:** 2011-06-30

**Authors:** Hajnalka Daligault, Alla Lapidus, Ahmet Zeytun, Matt Nolan, Susan Lucas, Tijana Glavina Del Rio, Hope Tice, Jan-Fang Cheng, Roxanne Tapia, Cliff Han, Lynne Goodwin, Sam Pitluck, Konstantinos Liolios, Ioanna Pagani, Natalia Ivanova, Marcel Huntemann, Konstantinos Mavromatis, Natalia Mikhailova, Amrita Pati, Amy Chen, Krishna Palaniappan, Miriam Land, Loren Hauser, Evelyne-Marie Brambilla, Manfred Rohde, Susanne Verbarg, Markus Göker, James Bristow, Jonathan A. Eisen, Victor Markowitz, Philip Hugenholtz, Nikos C. Kyrpides, Hans-Peter Klenk, Tanja Woyke

**Affiliations:** 1 DOE Joint Genome Institute, Walnut Creek, California, USA; 2 Los Alamos National Laboratory, Bioscience Division, Los Alamos, New Mexico, USA; 3 Biological Data Management and Technology Center, Lawrence Berkeley National Laboratory, Berkeley, California, USA; 4 Oak Ridge National Laboratory, Oak Ridge, Tennessee, USA; 5 DSMZ - German Collection of Microorganisms and Cell Cultures GmbH, Braunschweig, Germany; 6 HZI – Helmholtz Centre for Infection Research, Braunschweig, Germany; 7 University of California Davis Genome Center, Davis, California, USA; 8 Australian Centre for Ecogenomics, School of Chemistry and Molecular Biosciences, The University of Queensland, Brisbane, Australia

**Keywords:** strictly aerobic, non-motile, Gram-negative, branching, sheathed, mesophilic, chemoorganotrophic, "*Saprospiraceae*", GEBA

## Abstract

*Haliscomenobacter hydrossis* van Veen *et al.* 1973 is the type species of the genus *Haliscomenobacter*, which belongs to order "*Sphingobacteriales*". The species is of interest because of its isolated phylogenetic location in the tree of life, especially the so far genomically uncharted part of it, and because the organism grows in a thin, hardly visible hyaline sheath. Members of the species were isolated from fresh water of lakes and from ditch water. The genome of *H. hydrossis* is the first completed genome sequence reported from a member of the family "*Saprospiraceae*". The 8,771,651 bp long genome with its three plasmids of 92 kbp, 144 kbp and 164 kbp length contains 6,848 protein-coding and 60 RNA genes, and is a part of the *** G****enomic* *** E****ncyclopedia of* *** B****acteria and* *** A****rchaea * project.

## Introduction

Strain O^T^ (= DSM 1100 = ATCC 27775) is the type strain of *Haliscomenobacter hydrossis* which is the type and only species within the genus *Haliscomenobacter* [1,2]. The generic name derives from the Greek word *haliskomai*, to be imprisoned, and the Neo-Latin *bacter*, a rod, meaning the imprisoned rod. The species epithet is derived from the Greek word *hudôr*, water, and *Oss*, a town in the Netherlands, *hydrossis*, from water of Oss. The imprisoned rod from the water of Oss. Five morphologically and physiologically congruent strains belonging to the species, including the type strain O^T^, were isolated from activated sludge samples in the early 1970s [1]. *H. hydrossis* was sporadically observed in aeration tanks of sewage treatment plants in Germany [3] and in paper industry wastewater treatment plants in France [4]. As a recent biotechnological application, biomass bulking caused by *H. hydrossis* was controlled by lytic bacteriophages [5]. An improved high quality draft sequence of *Saprospira grandis* strain Sa g1 (=HR1, DSM 2844, GOLD ID Gi033955) is the only other genomic information currently available from the family "*Saprospiraceae*". Here we present a summary classification and a set of features for *H. hydrossis* O^T^, together with the description of the complete genomic sequencing and annotation.

## Classification and features

The single genomic 16S rRNA sequence of *H. hydrossis* O^T^ was compared using NCBI BLAST [6] under default settings (e.g., considering only the high-scoring segment pairs (HSPs) from the best 250 hits) with the most recent release of the Greengenes database [7] and the relative frequencies of taxa and keywords (reduced to their stem [8]) were determined, weighted by BLAST scores. The most frequently occurring genera were *Haliscomenobacter* (83.9%) and *Lewinella* (16.1%) (3 hits in total). Regarding the two hits to sequences from members of the species, the average identity within HSPs was 99.2%, whereas the average coverage by HSPs was 98.1%. Among all other species, the one yielding the highest score was *Lewinella antarctica* (EF554367), which corresponded to an identity of 89.1% and an HSP coverage of 66.6%. (Note that the Greengenes database uses the INSDC (= EMBL/NCBI/DDBJ) annotation, which is not an authoritative source for nomenclature or classification.) The highest-scoring environmental sequence was AJ786323 ('Lake Wolfgangsee freshwater enrichment clone MS-Wolf2-H'), which showed an identity of 98.8% and an HSP coverage of 97.9%. The most frequently occurring keywords within the labels of environmental samples which yielded hits were 'lake' (10.6%), 'tin' (5.3%), 'microbi' (3.4%), 'freshwat' (3.2%) and 'mat' (3.2%) (247 hits in total). The most frequently occurring keywords within the labels of environmental samples which yielded hits of a higher score than the highest scoring species were 'lake' (11.1%), 'tin' (5.6%), 'microbi' (3.5%), 'freshwat' (3.4%) and 'mat' (3.3%) (225 hits in total). These keywords reflect the ecological properties reported for strain O^T^ in the original description [1].

**Figure 1:**
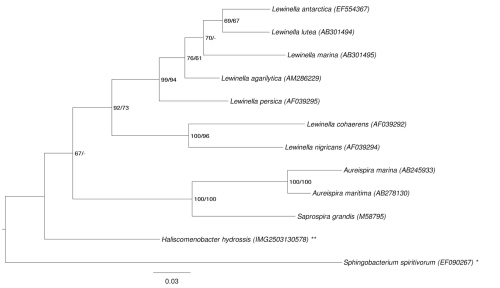
Phylogenetic tree highlighting the position of *H. hydrossis* relative to the type strains of the other species within the family "*Saprospiraceae*". The tree was inferred from 1,399 aligned characters [9,10] of the 16S rRNA gene sequence under the maximum likelihood (ML) criterion [11]. Rooting was done initially using the midpoint method [12] and then checked for its agreement with the current classification (Table 1). The branches are scaled in terms of the expected number of substitutions per site. Numbers adjacent to the branches are support values from 150 maximum likelihood bootstrap replicates [13] (left) and from 1,000 maximum parsimony bootstrap replicates [14] (right) if larger than 60%. Lineages with type strain genome sequencing projects registered in GOLD [15] are labeled with one asterisk, those also listed as 'Complete and Published' with two asterisks.

Figure 1 shows the phylogenetic neighborhood of *H. hydrossis* in a 16S rRNA based tree. The sequences of the two 16S rRNA gene copies in the genome differ from each other by two nucleotides and do not differ from the previously published 16S rRNA sequence AJ784892, which contains two ambiguous base calls.

**Table 1:** Classification and general features of *H. hydrossis* O^T^ according to the MIGS recommendations [16] and the NamesforLife database [17].

**MIGS ID**	**Property**	**Term**	**Evidence code**
	Current classification	Domain *Bacteria*	TAS [18]
		Phylum *Bacteroidetes*	TAS [19]
		Class ‘*Sphingobacteria*’	TAS [20]
		Order ‘*Spingobacteriales*’	TAS [20]
		Family ‘*Saprospiraceae’*	TAS [21]
		Genus *Haliscomenobacter*	TAS [1,2]
		Species *Haliscomenobacter hydrossis*	TAS [1]
		Type strain O	TAS [1,2]
	Gram stain	negative	TAS [1]
	Cell shape	rod-shaped with a hyaline sheath	TAS [1]
	Motility	non-motile	TAS [1]
	Sporulation	not reported	
	Temperature range	8–30°C	TAS [1]
	Optimum temperature	26°C	TAS [1]
	Salinity	not reported	
MIGS-22	Oxygen requirement	strictly aerobic	TAS [1]
	Carbon source	organic acids, peptides, proteins, mono- and polysaccharides	TAS [1]
	Energy metabolism	chemoorganotroph	TAS [1]
MIGS-6	Habitat	fresh water of lakes, ditch water	TAS [1]
MIGS-15	Biotic relationship	free-living	TAS [1]
MIGS-14	Pathogenicity	none	NAS
	Biosafety level	1	TAS [22]
	Isolation	bulking activated sludge	TAS [1]
MIGS-4	Geographic location	Oss, The Netherlands	TAS [1]
MIGS-5	Sample collection time	before 1973	TAS [1]
MIGS-4.1	Latitude	51.77	NAS
MIGS-4.2	Longitude	5.53	NAS
MIGS-4.3	Depth	0, surface	TAS [1]
MIGS-4.4	Altitude	about 8 m	NAS

Evidence codes - IDA: Inferred from Direct Assay (first time in publication); TAS: Traceable Author Statement (i.e., a direct report exists in the literature); NAS: Non-traceable Author Statement (i.e., not directly observed for the living, isolated sample, but based on a generally accepted property for the species, or anecdotal evidence). These evidence codes are from of the Gene Ontology project [23]. If the evidence code is IDA, the property was directly observed by one of the authors or an expert mentioned in the acknowledgements.

**Figure 2:**
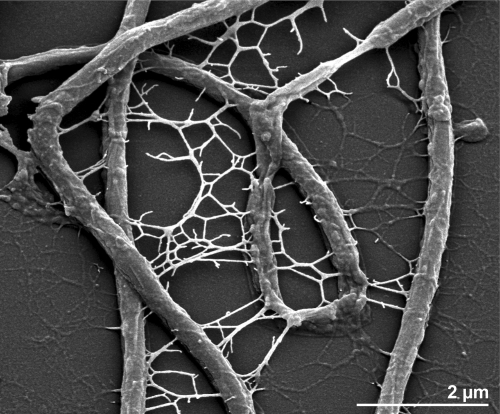
Scanning electron micrograph of *H. hydrossis* O^T^

The cells of *H. hydrossis* are rod-shaped, 0.35 – 0.45 µm wide and 3 - 5 µl long, mostly occurring in chains and nearly always enclosed by a narrow hyaline sheath (Figure 2) [1]. The sheath is sometimes disrupted by branching cells [1]. Flagella were never visible in EM images nor was motility ever observed [1]. Growing bacteria excrete so far unidentified polysaccharides [1]. Strain O^T^ grows strictly aerobically and produces intracellular carotenoid pigments [1]. Optimal growth temperature was 26°C, with a span of 8-30°C [1]. Optimal pH for growth was 7.5 [1]. Organic acids, peptides, proteins, mono- and polysaccharides were reported as carbon and energy sources [1]. Starch and gelatine were decomposed by all strains of the species [1], sorbitol, glycerol, lactate, acetate, succinate and β-hydroxybutyrate were not utilized [1]. The authors of the original description of the strain suggested that O^T^ accumulates polysaccharides either intra- or extracellularily [1].

### Chemotaxonomy

Nothing is known about the structure of the cell wall of *H. hydrossis*. The six major fatty acids of strain O^T^ were *iso*-C_15:0 3-OH_ (22.8%), *iso*-C_15:0_ (21.0%), C_16:1_ (17.3%), *iso*-C_15:0 2-OH_ (15.5%), and C_18:0_ (6.9%) and C_16:0_ (5.7%) [24]. The type strain contained significantly more hydroxylated fatty acids than several analyzed reference strains from the genus [24]. Observed quinones were mainly of the MK-7 type (70-90%), with 10-30% MK-6 [24].

## Genome sequencing and annotation

### Genome project history

This organism was selected for sequencing on the basis of its phylogenetic position [25], and is part of the *** G****enomic* *** E****ncyclopedia of* *** B****acteria and* *** A****rchaea * project [26]. The genome project is deposited in the Genome On Line Database [15] and the complete genome sequence is deposited in GenBank. Sequencing, finishing and annotation were performed by the DOE Joint Genome Institute (JGI). A summary of the project information is shown in Table 2.

**Table 2:** Genome sequencing project information

**MIGS ID**	**Property**	**Term**
MIGS-31	Finishing quality	Finished
MIGS-28	Libraries used	Four genomic libraries: 454 pyrosequence standard library, 454 PE libraries (8 kb and 13 kb insert size), one Illumina library
MIGS-29	Sequencing platforms	Illumina GAii, 454 GS FLX Titanium
MIGS-31.2	Sequencing coverage	165.3 x Illumina; 38.5 x pyrosequence
MIGS-30	Assemblers	Newbler version 2.3, Velvet version 0.7.63, phrap version SPS - 4.24
MIGS-32	Gene calling method	Prodigal 1.4, GenePRIMP
	INSDC ID	CP002691 (chromosome) CP002692 (plasmid pHALHY01) CP002693 (plasmid pHALHY02) CP002694 (plasmid pHALHY03)
	Genbank Date of Release	May 9, 2011
	GOLD ID	Gc01752
	NCBI project ID	48289
	Database: IMG-GEBA	2504756004
MIGS-13	Source material identifier	DSM 1100
	Project relevance	Tree of Life, GEBA

### Growth conditions and DNA isolation

*H. hydrossis* O^T^, DSM 1100, was grown in DSMZ medium 134 (*Haliscomenobacter* Medium) [27] at 26°C. DNA was isolated from 0.5-1 g of cell paste using MasterPure Gram-positive DNA purification kit (Epicentre MGP04100) following the standard protocol as recommended by the manufacturer, with modification st/DL for cell lysis as described in Wu *et al*. [26]. DNA is available through the DNA Bank Network [28].

### Genome sequencing and assembly

The genome was sequenced using a combination of Illumina and 454 sequencing platforms. All general aspects of library construction and sequencing can be found at the JGI website [29]. Pyrosequencing reads were assembled using the Newbler assembler (Roche). The initial Newbler assembly consisting of 153 contigs in three scaffolds was converted into a phrap [30] assembly by making fake reads from the consensus, to collect the read pairs in the 454 paired end library. Illumina GAii sequencing data (1,273.3 Mb) was assembled with Velvet [31] and the consensus sequences were shredded into 1.5 kb overlapped fake reads and assembled together with the 454 data. The 454 draft assembly was based on 369.3 Mb 454 draft data and all of the 454 paired end data. Newbler parameters are -consed -a 50 -l 350 -g -m -ml 20. The Phred/Phrap/Consed software package [30] was used for sequence assembly and quality assessment in the subsequent finishing process. After the shotgun stage, reads were assembled with parallel phrap (High Performance Software, LLC). Possible mis-assemblies were corrected with gapResolution [29], Dupfinisher [32], or sequencing cloned bridging PCR fragments with subcloning. Gaps between contigs were closed by editing in Consed, by PCR and by Bubble PCR primer walks (J.-F. Chang, unpublished). A total of 589 additional reactions were necessary to close gaps and to raise the quality of the finished sequence. Illumina reads were also used to correct potential base errors and increase consensus quality using a software Polisher developed at JGI [33]. The error rate of the completed genome sequence is less than 1 in 100,000. Together, the combination of the Illumina and 454 sequencing platforms provided 203.8 × coverage of the genome. The final assembly contained 1,005,536 pyrosequence and 35,370,321 Illumina reads.

### Genome annotation

Genes were identified using Prodigal [34] as part of the Oak Ridge National Laboratory genome annotation pipeline, followed by a round of manual curation using the JGI GenePRIMP pipeline [35]. The predicted CDSs were translated and used to search the National Center for Biotechnology Information (NCBI) non-redundant database, UniProt, TIGR-Fam, Pfam, PRIAM, KEGG, COG, and InterPro databases. Additional gene prediction analysis and functional annotation was performed within the Integrated Microbial Genomes - Expert Review (IMG-ER) platform [36].

## Genome properties

**Figure 3:**
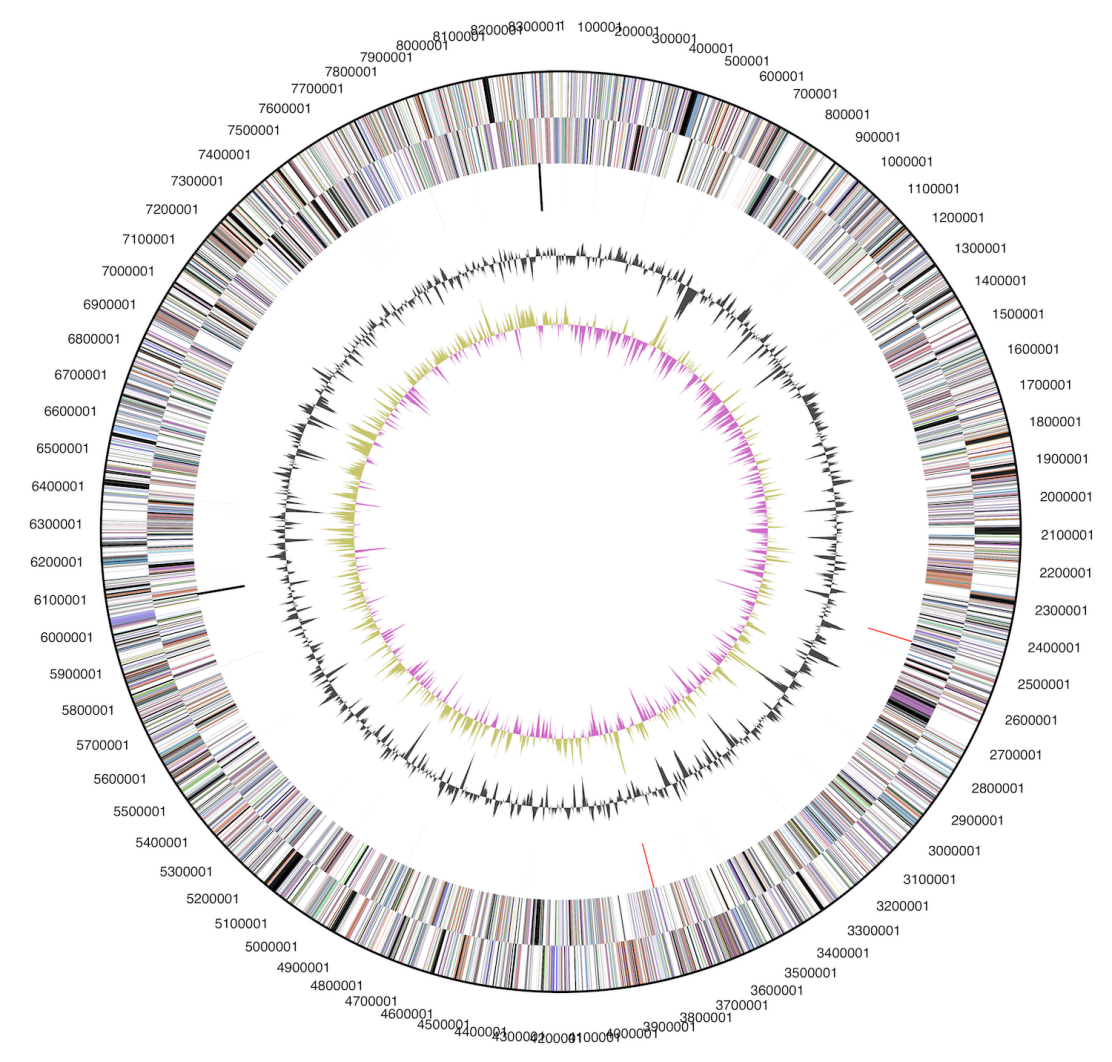
Graphical circular map of the chromosome (plasmid maps not shown). From outside to the center: Genes on forward strand (color by COG categories), Genes on reverse strand (color by COG categories), RNA genes (tRNAs green, rRNAs red, other RNAs black), GC content, GC skew.

The genome consists of an 8,371,686 bp long circular chromosome and three plasmids of 164,019 bp, 143,757 bp and 92,189 bp length, respectively, with a G+C content of 47.1% (Table 3 and Figure 3). Of the 6,918 genes predicted, 6,858 were protein-coding genes, and 60 RNAs; 106 pseudogenes were also identified. The majority of the protein-coding genes (58.6%) were assigned with a putative function while the remaining ones were annotated as hypothetical proteins. The distribution of genes into COGs functional categories is presented in Table 4.

**Table 3:** Genome Statistics

**Attribute**	**Value**	**% of Total**
Genome size (bp)	8,771,651	100.00%
DNA coding region (bp)	7,756,096	88.42%
DNA G+C content (bp)	4,131,717	47.10%
Number of replicons	4	
Extrachromosomal elements	3	
Total genes	6,918	100.00%
RNA genes	106	0.87%
rRNA operons	2	
Protein-coding genes	6,858	99.13%
Pseudo genes	106	1.53%
Genes with function prediction	4,054	58.60%
Genes in paralog clusters	325	4.70%
Genes assigned to COGs	3,905	56.45%
Genes assigned Pfam domains	4,456	64.41%
Genes with signal peptides	2,889	41.76%
Genes with transmembrane helices	1,588	22.95%
CRISPR repeats	8	

**Table 4:** Number of genes associated with the general COG functional categories

**Code**	**value**	**% age**	**Description**
J	171	4.0	Translation, ribosomal structure and biogenesis
A	0	0.0	RNA processing and modification
K	349	8.2	Transcription
L	190	4.4	Replication, recombination and repair
B	2	0.1	Chromatin structure and dynamics
D	26	0.6	Cell cycle control, cell division, chromosome partitioning
Y	0	0.0	Nuclear structure
V	146	3.4	Defense mechanisms
T	291	6.8	Signal transduction mechanisms
M	333	7.8	Cell wall/membrane/envelope biogenesis
N	20	0.5	Cell motility
Z	0	0.0	Cytoskeleton
W	0	0.0	Extracellular structures
U	64	1.5	Intracellular trafficking, secretion, and vesicular transport
O	161	3.8	Posttranslational modification, protein turnover, chaperones
C	216	5.1	Energy production and conversion
G	271	6.3	Carbohydrate transport and metabolism
E	306	7.2	Amino acid transport and metabolism
F	79	1.9	Nucleotide transport and metabolism
H	150	3.5	Coenzyme transport and metabolism
I	133	3.1	Lipid transport and metabolism
P	254	5.9	Inorganic ion transport and metabolism
Q	95	2.2	Secondary metabolites biosynthesis, transport and catabolism
R	596	13.9	General function prediction only
S	423	9.9	Function unknown
-	3,013	43.6	Not in COGs
